# Inhibition of Embryonic HSP 90 Function Promotes Variation of Cold Tolerance in Zebrafish

**DOI:** 10.3389/fgene.2020.541944

**Published:** 2020-12-04

**Authors:** Bingshe Han, Juntao Luo, Penglei Jiang, Yan Li, Qiong Wang, Yajing Bai, Jing Chen, Jian Wang, Junfang Zhang

**Affiliations:** ^1^Key Laboratory of Exploration and Utilization of Aquatic Genetic Resources Ministry of Education, Shanghai Ocean University, Shanghai, China; ^2^International Research Center for Marine Biosciences, Ministry of Science and Technology, Shanghai Ocean University, Shanghai, China; ^3^National Demonstration Center for Experimental Fisheries Science Education, Shanghai Ocean University, Shanghai, China

**Keywords:** Hsp90, zebrafish, cold tolerance, embryonic, Hsp90 inhibitor

## Abstract

Accumulating evidence indicates that heat shock protein 90 (HSP90) plays essential roles in modulation of phenotypic plasticity in vertebrate development, however, the roles of HSP90 in modulation of cold tolerance capacity in fish are still unclear. In the present study, we showed that transient inhibition of embryonic HSP90 function by a chemical inhibitor or low conductivity stress promoted variation of cold tolerance capacity in adult zebrafish. Further work showed that embryonic HSP90 inhibition enhanced cold tolerance in adult zebrafish could be transmitted to their offspring. RNA-seq data showed that embryonic HSP90 inhibition enhanced cold tolerance involves variation of gene expression related to proteasome, lysosome, autophagy, and ribosome. Experiments with zebrafish ZF4 cells showed that two differentially expressed genes *atg9b* and *psmd12* were up-regulated by radicicol treatment and provided protective roles for cells under cold stress, indicating that up-regulation of autophagy and proteasome function contributes to enhanced cold tolerance. The present work sheds a light on the roles of HSP90 in regulation of phenotypic plasticity associated with thermal adaptation in fish.

## Introduction

Heat shock proteins (HSPs) are a family of stress proteins that are expressed in prokaryote and eukaryote cells and tissues both constitutively and in response to biotic and abiotic stressors, acting as molecular chaperones that protect the cell against denatured proteins (Terasawa et al., 2005). HSPs are organized by molecular mass: for example HSP90 (85–90 kDa), HSP70 (68–73 kDa) and low-molecular-mass proteins (16–47 kDa) (Terasawa et al., 2005). It is well accepted that HSP90 helps correct folding of nascent proteins, refolding of misfolded proteins, and removal of incorrigibly misfolded proteins. HSP90 is required for the maturation, stability, degradation of over 400 client proteins, which are involved in multiple cellular functions including signal transduction, DNA replication, and biosynthesis (Taipale et al., 2010; Samant et al., 2012).

Heat shock proteins are thought to play a role in long-term adaptation to extended periods of environmental stress (Zabinsky et al., 2019), HSPs increase after the initial exposure to stress and protect tissues from structural damage during subsequent exposures (Hoekstra et al., 1998; Lei et al., 2009; Li and Mak, 2009). In addition to their immediate action after stress exposure, compromised HSP90 activity during development results in generation of novel phenotypes. Impaired HSP90 function in Drosophila failed to buffer the pre-existing cryptic genetic variants, and the expression of these genetic variants resulted in various morphological abnormalities (Rutherford and Lindquist, 1998). Further study showed that impaired HSP90 function in Drosophila led to the induction of morphological mutants via transposon activation (Specchia et al., 2010). Although the relationship between HSP90 inhibition and generation of new phenotypes has been documented in various species (Yeyati et al., 2007; Chen et al., 2012; Karras et al., 2017), how HSP90 modulates the ability of fishes to survive at low temperatures has not been reported so far.

Zebrafish (Danio rerio) is a major model system widely used for studies of development, disease and other biological processes. Zebrafish can survive a wide range of temperature of 16∼38°C (Engeszer et al., 2007), making itself an ideal model for study of thermal adaptation and acclimation. Study of cold acclimation or response in zebrafish has revealed characteristic cold adaptive variation of transcriptome and methylome (Long et al., 2012, 2013; Han et al., 2016), suggesting the existence of variable cold adaptive phenotypes.

In the present study, zebrafish embryos were subjected to a transient treatment with a HSP90 chemical inhibitor radicicol, and increased variation of cold tolerance capacity of adult zebrafish was observed. Further work showed that HSP90 inhibition enhanced cold tolerance in adult zebrafish could be transmitted to the next generation. RNA-seq data showed that HSP90 inhibition enhanced cold tolerance involved variation of transcriptome related to proteasome, lysosome, autophagy, and ribosome. Among the differentially expressed genes, up-regulation of *atg9b* and *psmd12* by radicicol was shown to protect zebrafish ZF4 cells under acute cold stress. Low conductivity condition also perturbed HSP90 function in zebrafish embryos and resulted in enhanced variation of cold tolerance capacity in adult zebrafish. Our data indicated that interference of HSP90 function during development by chemical inhibitor or environmental stress promotes variation of cold tolerance in zebrafish.

## Materials and Methods

### Zebrafish Maintenance and Treatment

The experimental protocol was approved by the Animal Ethics committee of Shanghai Ocean University and abides by the Guidelines on Ethical Treatment of Experimental Animals established by the Ministry of Science and Technology, China. Zebrafish were maintained and staged according to standard methods (Kimmel et al., 1995). The wild-type (AB) zebrafish was used for all experiments. Breeding fish were maintained at 28°C in a circulating water system on a 14-h light/10-h dark cycle. Embryos were collected by natural spawning and staged.

For treatment, about 100 embryos collected from breeding pairs were incubated in system water at 28°C, and each spawning was split into two groups. A half of embryos were treated, while the other half was kept as control. Treatment with 5 μM radicicol (553400, Sigma, dissolved in DMSO at 5 mM) was initiated at 50% epiboly and continued to 48 hpf, followed by the removal of the drug using three rinses, the whole process was carried in the dark because the light sensitivity of radicicol. Control group were treated with identical doses of DMSO. Each experiment had three biological replicates.

For cold treatment, 3-month-old zebrafish from radicicol and control group were used. To avoid variability between experiments, each group have equal zebrafish about 25∼30 tails in fish tank with 10 L of system water, which was separated with fine meshed Fishnet in the middle. Then the two groups were subjected to a stepwise cooling process at a rate of ∼1°C/h to 18°C by strict temperature control in a low temperature incubator (LY-36VL, Percival Scientific). The fishes were maintained at 18°C for 12 h, then subjected to another stepwise cooling process at a rate of ∼1°C/h to 8°C. Each experiment had three biological replicates. The death was defined when zebrafish experienced a loss of equilibrium and entered a coma. The survival time was calculated from the beginning of cold treatment to the time of death.

### Immunoblot and Antibodies

After treatment, fishes or embryos were washed twice with phosphate-buffered saline (PBS), drained, cut into small pieces, and 0.5 ml of lysis buffer (20 mM Tris-HCl, pH 7.5, 150 mM NaCl, 1 mM Na_2_EDTA, 1 mM EGTA, 1% Triton X-100, 2.5 mM sodium pyrophosphate, 1 mM β-glycerophosphate, 1 mM Na_3_VO_4_ and 1 μg/ml leupeptin) supplemented with protease inhibitors was added to each 100 mg of tissue. After 5 min on ice, the samples were sonicated. After centrifugation at 4°C and 15,000 × *g* for 10 min, protein aliquots containing 30 μg of protein were separated on denaturing and reducing Laemmli 12% polyacrylamide gels and transferred to nitrocellulose membrane. The membrane was blocked in PBS containing 5% milk powder and 0.1% Tween 20, and incubated at 4°C overnight with primary antibody and for 1 h at 25°C with horseradish peroxidase-conjugated secondary antibody. Antibody binding was visualized using chemiluminescence detection reagent with Amersham Imager 600. Antibodies: Rabbit anti-HSP90 (ab13495, Abcam); Mouse anti-β-Actin (A1978, Sigma), HRP-linked goat anti-rabbit IgG (7074S, Cell Signaling Technology).

### Quantitative RT-PCR (qRT-PCR)

Total RNA was isolated using TRlzol reagent (15596-026, Life Technologies) following standard protocol. One microgram of total RNA was reverse-transcribed to cDNA using QuantiTect reverse transcription kit (205311, Qiagen). Gene expression was measured by quantitative PCR using Roche LightCycler 480 System and LightCycler 480 SYBR Green I Master (04707516001, Roche). RNA level was normalized to glyceraldehyde-3-phosphate dehydrogenase (GAPDH) mRNA level. Relative mRNA level was analyzed by the comparative CT method. The primers designed to detect the target genes are shown in Supplementary Table S1.

### Library Construction and High-Throughput Sequencing

Three zebrafish from radicicol group showing significantly increased survival times under cold stress and three zebrafish from control group were selected for RNA-seq, 3 replicates in each group. Muscle tissue was separated and used as materials for RNA-seq. Total RNA was extracted using TRlzol reagent (15596-026, Life Technologies) following the manufacturer’s instructions and checked for a RIN number to inspect RNA integrity by an Agilent Bioanalyzer 2100 (Agilent Technologies). Qualified total RNA was further purified by RNAClean XP Kit (A63987, Beckman Coulter) and RNase-Free DNase Set (79254, Qiagen). Total 6 libraries were constructed using VAHTS Total RNA-seq (H/M/R) Library PrepKit for Illumina (NR603-02, Vazyme). Libraries were pooled and sequenced using the Illumina HiSeq machine as 150-bp paired-end sequencing reads.

### Bioinformatic Analysis of RNA-seq Data

The raw reads were trimmed and filtered using Seqtk^1^. Low quality (*Q* < 20) bases were trimmed from 3′ ends of the reads and the trimmed reads were filtered with read length ≥ 25 bp. Clean RNA-seq reads for each sample were aligned by HISAT2 (2.0.4) with default setting to the zebrafish genome assembly using the Ensembl annotation DanRer10 (Danio_rerio.GRCz10.84.gtf) (Kim et al., 2015). The number of reads mapped to the genes was counted by StringTie and normalized by TMM (trimmed mean of *M* values) method (Robinson and Oshlack, 2010). We next calculated fragments per kilobase per million mapped reads (FPKM) of each gene to indicate gene expression level. Genes with low read counts (count-per-million < 1, at least in 3 samples) were filtered out before differentially expression analysis. Fisher’s exact test was then used to identify differentially expressed genes (DEGs) by edger with a fold change > 2 and *P*-value < 0.05(Robinson et al., 2010).

### GO and KEGG Pathway Enrichment Analyses

Database for Annotation, Visualization and Integrated Discovery (DAVID) v6.8 web tool^2^ were used to perform GO and KEGG enrichment analyses with a significance of *P* < 0.05 (Huang da et al., 2009; Huang et al., 2009).

### Cell Culture and Treatment

ZF4 cell line was purchased from the American Type Culture Collection (ATCC, Cat No. CRL2050). The cells were grown at 28°C, 5% CO_2_, in Dulbecco’s modified Eagle’s medium/F12 nutrient mix (SH30023.01B, Hyclone, Thermo Scientific) supplemented with 10% fetal bovine serum (10099141, Gibco, Life technologies), 1% penicillin-streptomycin-glutamine solution (SV30082.01, Hyclone, Thermo Scientific).

For cold treatment, ZF4 cells were seeded at 40–50% confluence and the next day moved into an incubator at 10°C, 5% CO_2_, in the same medium. For cell viability assay, cells were prepared into a cell suspension and stained with 0.4% trypan blue solution (w/v) for 5 min, then counted with a hemocytometer. All experiments were performed in triplicate.

### shRNA Lentivirus Production and Cell Transduction

DNA oligos for zebrafish *atg9b* and *psmd12* genes (Supplementary Table S1) were synthesized by Shanghai Sangon Biotechnology (Shanghai, China). After annealing, the double-strand DNA was cloned into the shRNA expressing vector pLKO.1-puro. In all, 3 μg of appropriate packaging plasmids pCMV-VSVG: pCMV-DR 8.91 (1: 5) and 5 μg of shRNA expressing vector were co-transfected into 2.5 × 10^6^ HEK-293T cells using PolyFect transfection reagent (301105; Qiagen). Seventy-two hours later, the media containing lentivirus particles were collected and centrifuged at 1500 × *g* for 10 min. Then the supernatant was collected and used to infect ZF4 cells immediately in the presence of 10 μg/ml hexadimethrine bromide (a.k.a. polybrene, H9268; Sigma-Aldrich). Forty-eight hours after infection, cells were collected for analysis.

### Motif Enrichment Analysis

We performed motif enrichment analysis to identify the key transcription factors (TFs) that drive gene expression up-regulated. The promoter sequences (−1000 bp, +500 bp) of up-regulated genes were used as input data and uploaded to online motif analysis tool- CentriMo (Bailey and Machanick, 2012). The significant preference motifs were identified with default parameter. The motif logo were plotted by R package motifStack (Ou et al., 2018).

### Statistics Analysis

Statistical analysis of survival time variance was performed in R program using two-sided *F* test. qRT-PCR statistical analysis was assessed using two tailed *t*-test with the GraphPad Prism 5 statistical software package. All data were shown as standard deviation (SD) of three independent experiments. *P*-value < 0.05 was regarded to be statistically significant. ^∗^*P* < 0.05, ^∗∗^*P* < 0.01, ^∗∗∗^*P* < 0.001.

## Results

### Radicicol Treatment Inhibits HSP90 Function in Zebrafish Embryos

**FIGURE 1:**
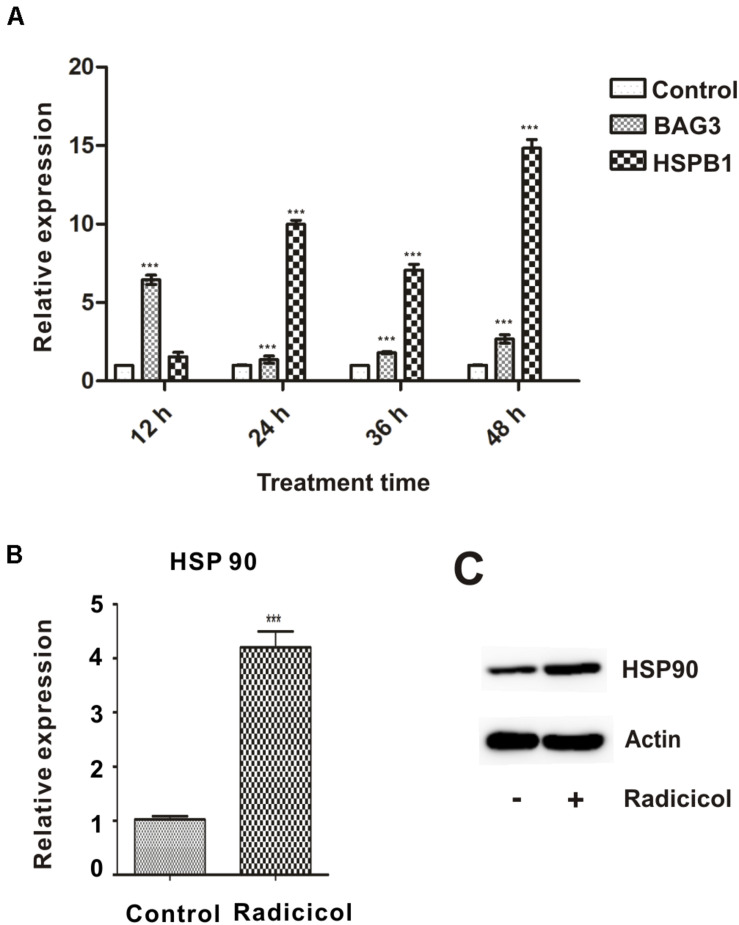
Inhibition of HSP90 function in zebrafish embryos by radicicol. **(A)** Zebrafish embryos were treated with 5 μM radicicol (radicicol group) or DSMO (control group) for indicated times, then the mRNA levels of BAG3 and HSPB1 were detected using qRT-PCR. **(B,C)** Zebrafish embryos were treated with 5 μM radicicol for 24 h, then HSP90 mRNA **(B)** and protein **(C)** levels were determined by qRT-PCR and immunoblot. *n* = 3, two tailed *t*-test, ***P < 0.001.

To compromise HSP90 function during embryonic development of the zebrafish, a well-characterized and highly specific inhibitor radicicol was used (Hadden et al., 2006). In our previous study, 5 μM radicicol treatment showed minor abnormal developmental traits and death, while 10 μM radicicol led to obvious developmental abnormalities and embryo death (Luo et al., 2017). So we chose 5 μM radicicol for further study, zebrafish embryos were treated with 5 μM radicicol from 50% epiboly to 48 hpf, then the inhibitor was removed. Inhibition of HSP90 function was confirmed by significantly increased expression of two marker genes *bag3*, *hspb1* (*hsp27*) for HSP90 inhibition, and HSP90 itself (Figures 1A–C), indicating the impairment of chaperone activity of HSP90 (Rohner et al., 2013). The results showed that 5 μM radicicol treatment inhibited HSP90 function effectively in zebrafish embryos.

### Inhibition of Embryonic HSP90 Function Promotes Variation of Cold Tolerance in Adult Zebrafish

**FIGURE 2:**
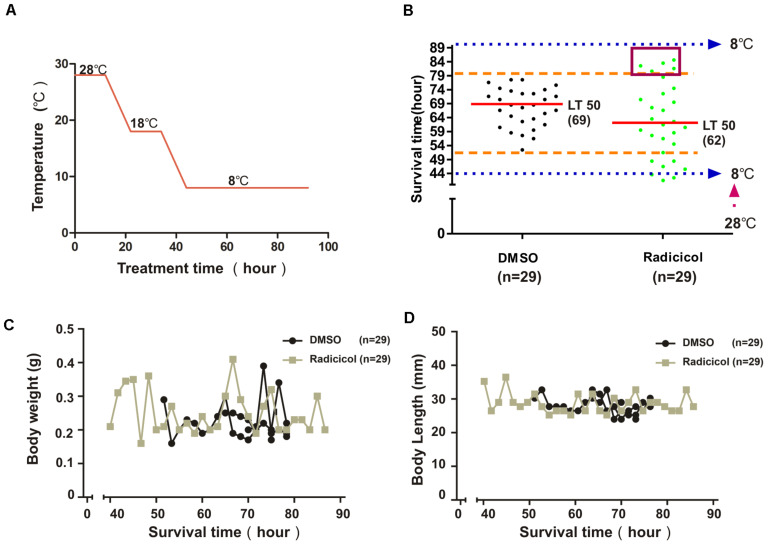
Impact of embryonic HSP90 inhibition on cold tolerance of zebrafish. **(A)** Schematic diagram of the cooling time course. The temperature decreased from 28 to 18°C at a rate of 1°C/h, remained at 18°C for 12 h (overnight), then decreased at a rate of 1°C/h until reaching a final temperature of 8°C. **(B)** Zebrafish from control and radicicol group were exposed to a temperature of 8°C according to the protocol in **(A)**, then survival time and the median lethal time (LT50) were measured. Dotted blue line (bottom) indicates the starting time at 8°C. Broken orange lines indicate the range of survival time of zebrafish of control group. Red box indicates zebrafish with increased survival time (cold tolerant zebrafish). **(C,D)** Body weight and length of fishes with different survival time for both groups.

To investigate the impact of embryonic HSP90 inhibition on cold tolerance of zebrafish, 3-month-old zebrafish from radicicol group and control group were exposed to cold stress. Figure 2A shows the process of cold exposure. Most zebrafish survived reaching a final temperature of 8°C except for several zebrafish from radicicol group (Figure 2B), indicating severely impaired cold tolerance capacity for these zebrafish, then the survival time of each zebrafish was recorded at 8°C. Compared with control group, we observed both significantly increased and decreased survival times in zebrafish from radicicol group (Figure 2B), which results in a statistically significant increase in the standard deviation (SD) of radicicol group compared with control group (two-sided *F* test, *P* = 0.0006). Meanwhile a decrease of the median lethal time (LT50) was observed in zebrafish from radicicol group (Figure 2B), suggesting globally impaired cold tolerance for radicicol group despite the fact that some zebrafish showed extended survival times. We observed no significant correlation between body weight or body length and survival time for both groups (Figures 2C,D), excluding the possibility that observed variation of cold tolerance is attributed to body weight. These data indicated that embryonic HSP90 inhibition released increased variation of cold tolerance capacity in zebrafish.

### HSP90 Inhibition Enhanced Cold Tolerance Is Inheritable

**FIGURE 3:**
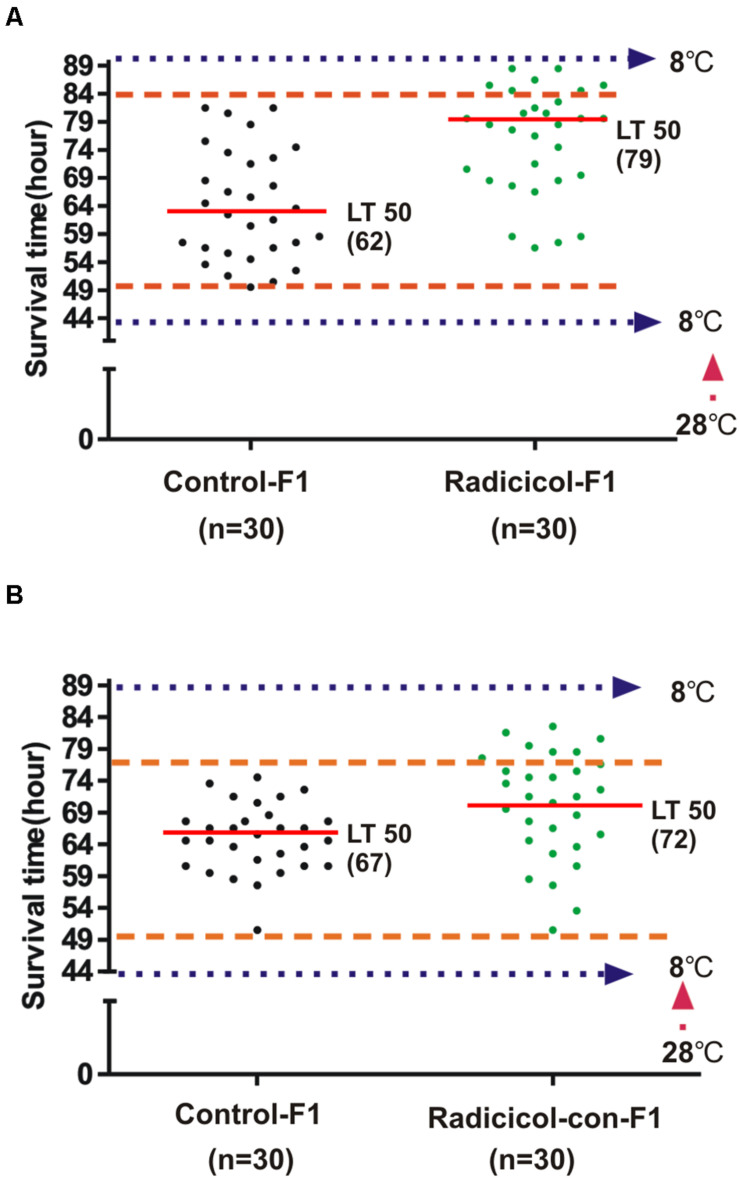
HSP90 inhibition enhanced cold tolerance is inheritable. **(A)** Three-month-old F1 zebrafish of cold tolerant parental fish (Radicicol-F1) and control F1 zebrafish (control) were exposed to cold pressure as in Figure 2A, then survival time and the median lethal time (LT50) were observed. **(B)** Three-month-old F1 zebrafish by breeding cold tolerant and control parental fish (Radicicol-con-F1) and control F1 zebrafish (control) were exposed to cold pressure as in Figure 2A, then survival time and the median lethal time (LT50) were observed.

Above results showed that embryonic HSP90 inhibition led to both significantly enhanced and impaired cold tolerance capacity. To investigate if these new traits are inheritable, we examined the zebrafish with enhanced cold tolerance from radicicol group (named as cold tolerant zebrafish, red boxed in Figure 2B), since these cold tolerant zebrafish were still alive when all zebrafish in control group died from cold stress (Figure 2B). Then we obtained F1 zebrafish of these cold tolerant zebrafish, meanwhile F1 zebrafish by the parental zebrafish from control group were also obtained. And 3-month-old F1 zebrafish were exposed to cold stress following the protocol in Figure 2A, and we observed that F1 zebrafish of cold tolerant parental zebrafish showed increased survival times accompanied by a significant increase (79 h vs. 62 h) in the median lethal time (LT50), compared with F1 zebrafish of control group (Figure 3A). Meanwhile, we also obtained F1 zebrafish by breeding between those cold tolerant zebrafish and control group zebrafish, the resulting F1 zebrafish also showed increased survival times (LT50: 72 h vs. 67 h), compared with F1 zebrafish of control group (Figure 3B). The data supported that HSP90 inhibition enhanced cold tolerance could be transmitted to the next generation.

### Variation of Gene Expression Pattern in Cold Tolerant Zebrafish

**FIGURE 4:**
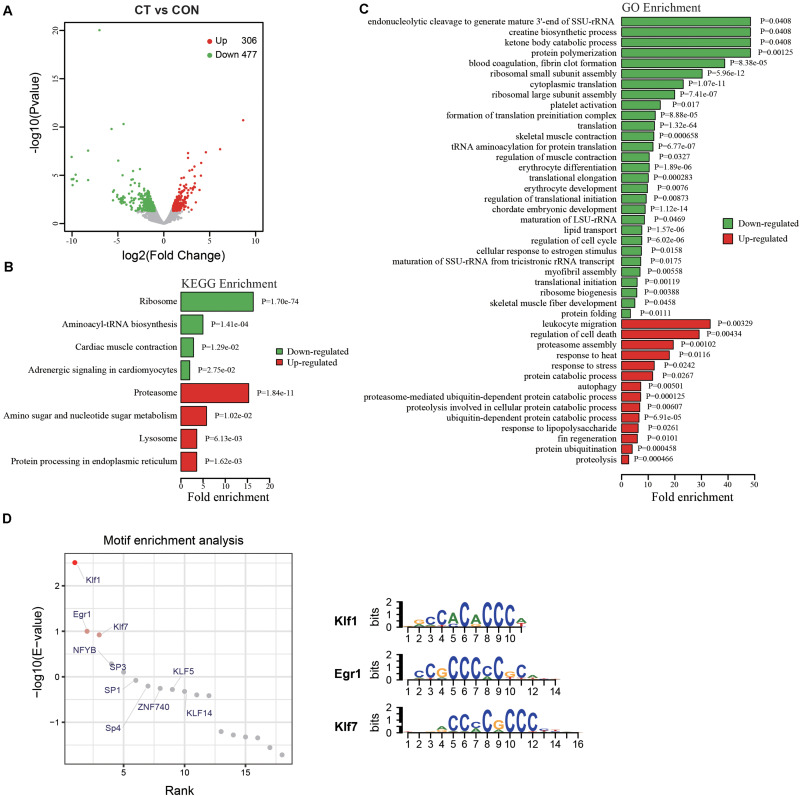
GO and KEGG pathway enrichment analyses of DEGs by RNA-seq. The total RNAs from the muscle tissues of three cold tolerant zebrafish from radicicol group (CT) and three control zebrafish (CON) were subjected to RNA-seq and subsequent analyses. **(A)** The Volcano plots of DEGs between cold tolerant (CT) and control (CON) fish. Abscissa represents log2 (fold-change), and ordinate represents –log10. Red dots denote the significantly up-regulated genes. Green dots denote the significantly down-regulated genes. Gray dots denote the non-differentially expressed genes. **(B,C)** GO and KEGG pathway enrichment analyses of up-regulated and down-regulated genes, respectively. Red bars represent up-regulated genes. Green bars represent down-regulated genes. Abscissa represents fold enrichment. **(D)** Motif enrichment analysis of up-regulated genes.

Most new traits are, at least partly, the results of altered gene expression pattern. To investigate the underlying mechanisms how embryonic HSP90 inhibition caused enhanced cold tolerance in adult zebrafish, RNA-seq was performed with the muscle tissues of cold tolerant zebrafish (CT) from radicicol group (red boxed in Figure 2B) after a one-month recovery from cold pressure, and the muscle tissues from untreated zebrafish from control group were used as control, with 3 replicates in each group. The numbers of raw reads were 157,068,718 and 163,472,400 for cold tolerant and control RNA libraries, respectively. After trimming low quality bases and filtering reads with length < 25 bp, we obtained 150,559,696 (control) and 155,605,275 (cold tolerant) high quality clean reads. Compared with control zebrafish (CON), a significantly varied expression profile was observed in cold tolerant zebrafish (CT), with 306 up-regulated and 477 down-regulated genes (Figure 4A and Supplementary Table S2). KEGG pathway analysis showed affected pathways including proteasome, lysosome, autophagy, and ribosome (Figure 4B and Supplementary Table S3). Gene Ontology analysis showed differentially expressed genes (DEGs) involved in protein synthesis and degradation, autophagy, and muscle development (Figure 4C and Supplementary Table S4). We also performed motif enrichment analysis to identify the key transcription factors (TFs) that drive gene expression up-regulated. Among the enriched motifs, we found highly significant enrichment for Klf1, Klf7 and Egr1 binding sites (Figure 4D and Supplementary Table S5). It is plausible that TFs binding to these sites play roles in up-regulation of genes. These data indicated HSP90 inhibition enhanced cold tolerance involves functional variation of protein synthesis and degradation, autophagy, and muscle development, and that these functional variation may contribute to enhanced cold tolerance in zebrafish.

### Validation and Function Analysis of Selected Differentially Expressed Genes

**FIGURE 5:**
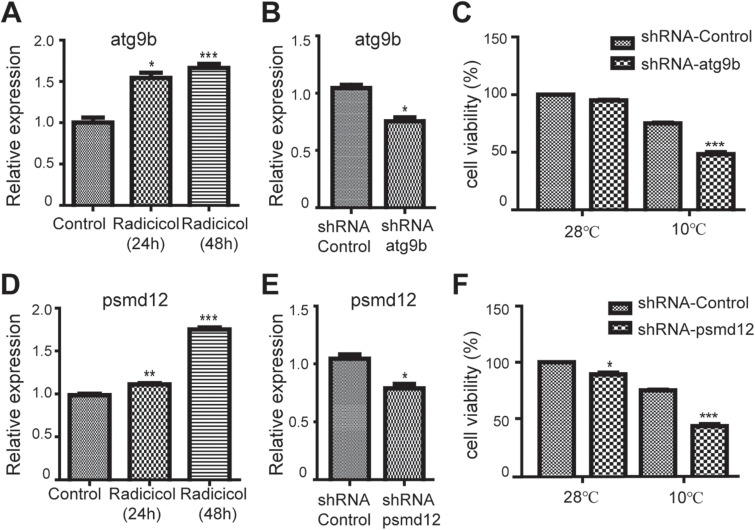
Roles of *atg9b* and *psmd12* in cold tolerance in zebrafish cells. **(A,D)** zebrafish ZF4 cells were treated with 5 μM radicicol for indicated times, then the mRNA levels of *atg9b* and *psmd12* were examined using qRT-PCR. **(B,E)** Zebrafish ZF4 cells were infected with shRNA lentivirus targeting *atg9b* or *psmd12* for 24 h, then the mRNA levels of *atg9b* and *psmd12* were examined using qRT-PCR. **(C,F)** Zebrafish ZF4 cells were infected with shRNA lentivirus targeting *atg9b* or *psmd12* for 24 h, then the cells were moved to an incubator at 10°C for 24 h, then cell viability was examined using trypan blue staining. All experiments were performed in triplicate. *n* = 3, two tailed *t*-test, ^∗^*p* < 0.05, ^∗∗^*p* < 0.01, ^∗∗∗^*p* < 0.001.

Above RNA-seq data showed the gene expression variation in cold tolerant zebrafish. The RNA-seq data were validated using qRT-PCR with six selected differentially expressed genes (Supplementary Figure S1), among which the roles of atg9b (autophagy-related protein 9b) and psmd12 (proteasome 26S Subunit, Non-ATPase 12) in cold tolerance were further studied using zebrafish ZF4 cells. ZF4 cells were infected with lentivirus expressing shRNA targeting *atg9b* or *psmd12*, cultured at 28°C for 24 h, and moved to an incubator at 10°C for cold treatment, then cell viability under this acute cold stress was examined. As expected, radicicol treatment could increase the expression of *atg9b* (Figure 5A) and *psmd12* (Figure 5D) in ZF4 cells, which is consistent with above RNA-seq data. Lentivirus infection could down-regulate the expression of *atg9b* (Figure 5B) and *psmd12* (Figure 5E). And *atg9b* shRNA (Figure 5C) and *psmd12* shRNA (Figure 5F) both decreased the cell viability of ZF4 cells under cold stress, indicating that HSP90 inhibition up-regulated *atg9b* and *psmd12* played protective roles in ZF4 cells under cold stress.

### Low Conductivity Stress During Embryonic Development Inhibits HSP90 and Promotes Variation of Cold Tolerance in Zebrafish

**FIGURE 6:**
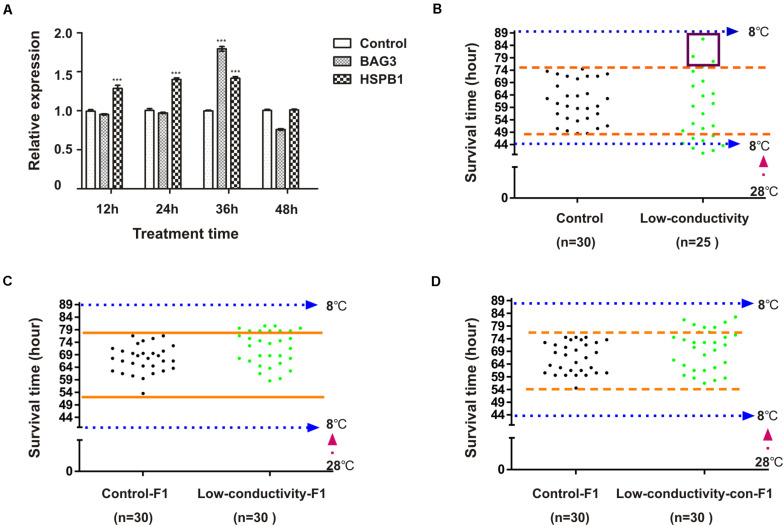
The impact of embryonic low-conductivity stress on cold tolerance of zebrafish. **(A)** Zebrafish embryos were incubated in low-conductivity water or regular embryo water for indicated times, the mRNA levels of BAG3 and HSPB1 were determined by qRT-PCR (*n* = 3, two tailed *t*-test, ^∗∗∗^*P* < 0.001). **(B)** Three-month-old zebrafish were subjected to cold pressure as in Figure 2A, then survival time was measured. **(C)** Three-month-old F1 zebrafish of cold tolerant parental fish (low-conductivity-F1) and control F1 zebrafish (control) were exposed to cold pressure as in Figure 2A, then survival time was observed. **(D)** Three-month-old F1 zebrafish by breeding cold tolerant and control parental fish (low-conductivity-con-F1) and control F1 zebrafish (control) were exposed to cold pressure as in Figure 2A, then survival time was observed.

Above results showed that embryonic radicicol treatment led to increased variation of cold tolerance in zebrafish, however whether HSP90 plays roles in modulation of cold tolerance in fish under natural conditions still remains unclear. It has been reported that low conductivity of surrounding water can compromised HSP90 function in cavefish (Rohner et al., 2013), here we also investigated the effect of low conductivity condition on the cold tolerance of zebrafish. Zebrafish embryos were incubated in de-ionized water (conductivity: 0.067 μs/cm) during 1–48 hpf, zebrafish embryos incubated in regular water (conductivity: 400 μs/cm) were used as control, then 3-month-old zebrafish were subjected to cold pressure as in Figure 2A. Inhibition of HSP90 was indicated by increased mRNA levels of *bag3* and *hspb1* (Figure 6A). Compared with control group, we also observed increased variation of survival times in the zebrafish from treatment group, which results in a statistically significant increase in the standard deviation (*SD*) (two-sided *F* test, *P* < 0.0001) (Figure 6B). Increased survival times were also observed in F1-zebrafish from cold tolerant parental zebrafish of treatment group, and in F1-zebrafish from breeding between cold tolerant zebrafish and control zebrafish, indicating the inheritance of HSP90 inhibition enhanced cold tolerance capacity (Figures 6C,D). Our data here suggested that low conductivity during embryonic stages interfered with HSP90 function and led to increased variation of cold tolerance in zebrafish.

## Discussion

Increasing evidence indicates that HSP90 plays an essential role in buffering the expression of cryptic variation, and that HSP90 inhibition induces the expression, inheritance and enrichment of abnormal phenotypes (Milton et al., 2006; Wong and Houry, 2006). Water temperature is an important factor for survival and evolution of fishes. Although some researches showed that HSPs are directly involved in thermal tolerance in fish and other species (Nakano and Iwama, 2002; Connolly and Hall, 2008; Stetina et al., 2015; Hassanpour et al., 2016; Stefanovic et al., 2016; Kelly et al., 2017), how embryonic HSP90 inhibition affects adaptive capacity to water temperature in zebrafish remains unclear.

As expected, radicicol treatment led to a strong increase of in the expression of *bag3* and *hspb1* in zebrafish embryos. Radicicol can competitively bind to the ADP/ATP binding pocket of HSP90 and inhibit its ATPase activity, leading to the inactivation of HSP90 chaperoning activity. Bag3 (BCL-2–associated athanogene 3) is a HSP70 co-chaperone, and Hspb1 (also called heat shock protein 27) is a member of the heat shock protein family. It has been observed that the expression of *bag3*, *hspb1* and HSP90 increases in response to inactivation of HSP90 chaperon activity, which makes *bag3* and *hspb1* two maker genes for HSP90 inhibition (Rohner et al., 2013).

In zebrafish from control group, we observed different survival times under cold stress, which reflects the existing variation of cold tolerance capacity in zebrafish caused mainly by natural genetic variation. Compared with control group, we observed significantly increased and decreased survival times in zebrafish from radicicol group, indicating that significantly increased and decreased cold tolerance capacity was released after embryonic radicicol treatment. The results showed that embryonic radicicol treatment facilitated the release of increased variation of cold tolerance capacity in zebrafish. It has been previously reported that unusually large variation in eye size in larval fish was observed when *A. mexicanus* surface fish were raised in the presence of radicicol (Rohner et al., 2013). These data support that HSP90 inhibition helps the release of new phenotypes in fish.

In the present work, we also observed enhanced cold tolerance in the offspring of cold tolerant parental fish from radicicol group (Figure 3). These findings indicated that embryonic HSP90 inhibition enhanced cold tolerance is persistent and inheritable in zebrafish. Considering the factors inhibiting HSP90 (chemical inhibitor and low conductivity stress) were removed at 48 hpf, some persistent and inheritable changes that happened during embryonic zebrafish must have been maintained in the adult zebrafish, and then been transmitted to their offspring. It has been also reported previously that incubation temperature during embryonic development has persistent effects on thermal acclimation capacity in zebrafish (Scott and Johnston, 2012), indicating a mechanism associated with genetic and epigenetic regulation. In mammalian cells HSP90 inhibition also causes durable changes, conferring a selective advantage under stress (Lawag et al., 2017). Above data indicates that HSP90 inhibition induces persistent and inheritable phenotypic plasticity via genetic/epigenetic mechanisms.

Usually genetic/epigenetic changes will lead to changes in gene expression, so we investigated the transcriptional variation related to HSP90 inhibition enhanced cold tolerance through RNA-seq. Analysis of the differentially expressed genes in the cold tolerant fish showed that ribosome function, protein synthesis, and muscle development were down-regulated, and that autophagy, lysosome, proteasome, and protein ubiquitination were upregulated. Repression of protein synthesis and enhanced protein folding and proteasome function have been also reported in the previous study about the gene expression variation induced by cold acclimation (Long et al., 2012; Scott and Johnston, 2012). We also noticed some characteristics of HSP90 inhibition enhanced cold tolerance such as up-regulated autophagy and the absence of strong variation of mitochondrial redox signaling.

Cell stress including cold stress will interfere with protein folding, and accumulation of misfolded proteins then overloads molecular chaperones including HSP90. Misfolded proteins can also be removed by proteolysis, which is mainly mediated by the ubiquitin (Ub)-proteasome system (UPS) and the autophagy-lysosome system (Gregersen and Bross, 2010). Several molecular chaperones such as the cochaperones CHIP (a cochaperone for HSP70 and HSP90) and BAG3 play important roles in decision making when targeting substrates for proteasomal or autophagic degradation (Ji and Kwon, 2017). Misfolded proteins lead to the generation of oxidative stress, which in turn induces cell death via damage of macromolecules such as DNA and protein. Autophagy plays essential roles in cell survival under various stress and contributes to fasting enhanced cold resistance in fish (Lu et al., 2019). Proteasome also plays roles in cells under stress (Stone, 2019), and proteasome inhibitors induce ROS production and apoptosis (Paniagua Soriano et al., 2014). The present study showed that *atg9b* and *psmd12*, which were up-regulated in cold tolerant fish, could play protective roles in ZF4 cells under cold stress. These data suggest that increased expression of *atg9b* or *psmd12* contribute to HSP90 inhibition enhanced cold tolerance, probably through up-regulation of autophagy and proteasome function (Wang et al., 2017; Du et al., 2020). And it has been reported that Atg9b up-regulates autophagy and inhibits apoptosis (Wang et al., 2017), and that *psmd12* encodes the non-ATPase subunit Psmd12 (aka RPN5) of the 19S regulator of 26S proteasome (Du et al., 2020). Meanwhile, suppressed protein synthesis also helps reduce misfolded proteins, ROS production and subsequent changes of mitochondrial redox signaling. Rpl22l1 (Ribosomal Protein L22 like 1) is a large ribosomal subunit protein and required for general protein synthesis (Zhang et al., 2017), down-regulation of Rpl22l1 indicated suppressed ribosomal large subunit assembly and protein synthesis in cold tolerant zebrafish.

Gene expression is likely to be regulated at the transcriptional level through programmed interactions between *cis*-regulatory elements and trans-factors (e.g., TFs). Here the motif enrichment analysis of up-regulated genes identified enriched motif binding sites of TFs, including Klf1, Klf7 and Egr1. Klf1 and Klf7 are members of Krüppel-like transcription factor family, Klf1 is an erythroid-enriched transcription factor and a critical regulator of erythropoiesis (Manwani and Bieker, 2014). It was also reported that KLF1 regulates genes involved in autophagy, cell cycle and mitosis (Magor et al., 2015). Klf7 acts as a negative regulator of adipocyte development and inhibits expression of adiponectin and insulin (Hsieh et al., 2019), Klf7 overexpression suppresses hematopoietic stem and progenitor cell function (Schuettpelz et al., 2012). Egr1 (Early growth response 1) is an immediate early transcriptional factor which acts as a coordinator of the complex response to stress, Egr1 controls the expression of a wide range of genes involved in metabolism, cell proliferation, and inflammation (Magee and Zhang, 2017). Egr1 also regulates the neuronal proteasome associated genes, including psmb9, SGK, and Tap1 (James et al., 2006). The analyses suggest these TFs may contribute to the regulation of autophagy and proteasome function in cold tolerant fish.

How HSP90 inhibition related expression variation was produced, maintained and transmitted remained unclear in this study. Considering all above phenomena happened in the absence of constant HSP90 inhibition, some existing mechanisms including regulation of transposon mobility (Ryan et al., 2016), buffering the effects of mammalian endogenous retroviruses (Hummel et al., 2017), and induction of aneuploidy (Chen et al., 2012) could be under consideration. Accumulating reports suggest that epigenetic mechanisms may play roles in this kind of process, since HSP90 induced phenotypes could be impaired by sodium butyrate, a chemical inhibitor for histone deacetylases (HDACs) (Sollars et al., 2003; Lawag et al., 2017). Some reports also showed interaction between HSP90 and multiple epigenetic enzymes including KDM4B (Ipenberg et al., 2013). So it is possible that embryonic HSP90 inhibition established variation of epigenetic modifications during embryonic development that resulted in inheritable gene expression variation in zebrafish. Further work will focus on global change of epigenetic modification associated with HSP90 inhibition to elucidate the underlying mechanism.

## Data Availability Statement

The datasets generated for this study can be found in the data used in this study has been deposited in NCBI’s Gene Expression Omnibus repository and are accessible through GEO accession number GSE111359.

## Ethics Statement

The animal study was reviewed and approved by Animal Ethics committee of Shanghai Ocean University.

## Author Contributions

BH and JZ designed the research. BH, JL, YL, QW, YB, JC, and JW performed the experiments. PJ and JZ analyzed the data. BH and JZ wrote the manuscript. All the authors have read and approved the manuscript for publication.

## Conflict of Interest

The authors declare that the research was conducted in the absence of any commercial or financial relationships that could be construed as a potential conflict of interest.

## Footnotes

1 https://github.com/lh3/seqtk

2 https://david.ncifcrf.gov/

## Supplementary Material

Supplementary Figure 1 Validation of RNA-seq data.

Supplementary Table 1 List of primers and oligos used for qRT-PCR and shRNA.

Supplementary Table 2 Differentially expressed genes between cold tolerant zebrafish from radicicol group (CT) and control zebrafish (CON) (fold > 2 and *P* < 0.05).

Supplementary Table 3 KEGG pathway enrichment data of differentially expressed genes (Fold > 2 and *P* < 0.05).

Supplementary Table 4 GO enrichment data of differentially expressed genes (Fold > 2 and *P* < 0.05).

Supplementary Table 5 Motif enrichment analysis of up-regulated genes.
