# Supplementary material for: Inhibition of Embryonic HSP 90 Function Promotes Variation of Cold Tolerance in Zebrafish
Source: Front Genet. 2020 Dec 4;11:541944. doi: 10.3389/fgene.2020.541944 (PMC7746879; doi:10.3389/fgene.2020.541944)
Supplement: Supplementary Figure 1 — Validation of RNA-seq data. [file Data_Sheet_1.PDF]

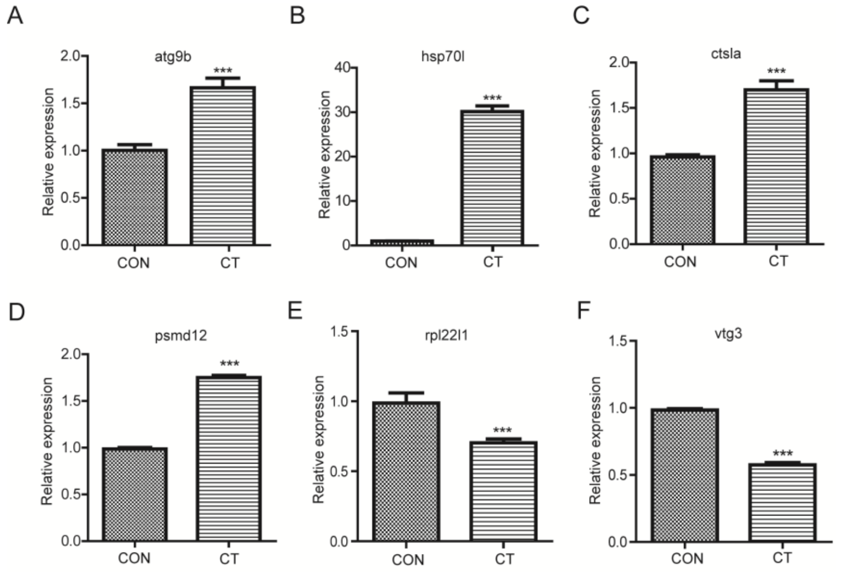

Supplementary Figure 1. Validation of RNA-seq data.

The total RNAs were isolated from the muscle tissues of three cold tolerant zebrafish from radicicol group (CT) and three control zebrafish (CON), then the mRNA levels of six selected differentilly expressed genes: atg9b, hsp70l, ctsla, psmd12, rpl22l1 and vtg3 were examined by qRT-PCR. N=3, two tailed t test, ** P<0.01; *** P<0.001.
